# A Buddhist Missionary

**Published:** 1888-02

**Authors:** 


### A BUDDHIST MISSIONARY.

It was a very remarkable face that instantly drew attention amid the stream of cabin passengers who descended the gangplank from the Cunard steamship Aurania. Unmistakably Oriental in every feature and yet intellectual in the highest degree ;

"A Buddhist touched with the burnished livery of the sun."

That was all the explanation the companion voyagers of this little man had to give to their curious friends. The passenger list said he was Mr. Chatterji, but Mr. Arthur W. Gebhard, of this city, who was awaiting him, called him Mr. Modini.

A luxuriant growth of hair, jet and glossy black, fringed a delicate cast of countenance, the eyes being the most striking feature. They were also black and had an intensity of expression that was almost startling. The nose was aquiline and small, but firm. When the gentleman lifted his curling mustache in a smile, which was not infrequent, he displayed teeth white and even.

Later in the day, after a short rest from ten days sea sickness, Modini Chatterji received a few friends at the residence of Mr. Gebhard, No. 27 West Fifty-first street. He appeared clothed in his native Hindoo costume, but the material was black. His figure is slight and full of nervous energy, and at times when aroused by the excitement of earnest conversation it vibrates in every muscle.

"I am nearly twenty-eight years old," he said, in the most fluent English, "and am a Brahmin by birth. I graduated from the University of Calcutta, but have passed my recent years in London and Paris. I belong to the Theosophical Society of Europe, but am not, strictly speaking, a Theosophist."

"How can that be?"

"Because I do not pretend to know what God is," said Mr. Chatterji, and then, without being apparently conscious that he was monopolizing the conversation, he began a monologue upon his conception of religion.

New York City has never before seen this young man. If he can be persuaded to lecture or to give from the platform the result of his meditations upon religion, there will be a revival of liberal thought that will justify his own words: "The world has never known an idea which did not emanate from the East."

While listening to the metaphysical subtleties and strongly original ideas of this youthful apostle from the land of the heathen, the ordinary man's mind becomes hopelessly entangled. All the time those speaking eyes rivet themselves upon the hearer so that he cannot falter in his attention.

"I have come to America for the first time at the invitation of my friends, but without any definite plans for the future," he said. "I have devoted my life to the study of religion in its purest form, and am prepared to say that the foundation of every creed is the same—a belief in one God."

Whether he had any independent income which enabled him to travel the world over, Mr. Chatterji refused to say. The object of life he conceived to be the extinction of sorrow. By nature every man wants to be active. No man can fold his hands and sleep. If that work, that expense of energy, is devoted toward the end of a livelihood, so much the better.

"Buddhism is not the true religion; neither is Christianity," he said.

"I conceive religion to be the highest possible development of a man's inner consciousness. Religion cannot be found in churches, because there the clergymen preach because they are paid for it. They seek to entertain rather than instruct, and dare not over-ride the sectarianism of their congregations any more than politicians dare to rebel against the principles of their constituents,"

Mr. Chatterji had observed that the American missionaries who went to evangelize his countrymen, met with no success whatever. It was simply because they sought to supplant a faith that was not superstious with one that was. In the census of India, taken in 1878, there was a numerical increase in number of converts to Christianity, but the Governor of Bengal expressed the opinion that this was anything rather than a matter for congratulation by the American missionary societies.

"It simply betrays," said the Governor, "a spirit of self-preservation of the natives, in that a few of them, and only a few comparatively, espouse the church of England because England rules them, and because without being nominally Christians they have no consideration in the courts."

Mr. Chatterji has conversed with the most acute theological minds in Europe upon the subject of religion, and so far from being converted to any particular creed has risen above all creeds, and believes that he has caught a glimpse of the coming universal religion that must eventually receive the whole world into a divine fellowship. He divides all good men into three classes: First, the prudent man; second, the moral man; third, the religious man.

The religious man of the future will not worship God, because that will place him below the Being, of whose existence the only proof he has is human consciousness. Mr. Chatterji proves the existence of God as he proves the existence of himself—"I think, therefore I am."

"When another man does you an injury," he said, "do not feel that you are suffering at the hands of an alien, but that the sensation of injury is the same as if you had bitten your tongue. All men are one in spirit and in turn one with God. It is only the religious man, however, who can experience or know this. Here are three fathers, and each feels toward his son an affection that is the same while differing in the object. That affection, that love, is God."

"He is a most extraordinary man," said Mr. Gebhard of his friend. "I met him in London two years ago, when he was creating the greatest kind of a stir in society. He will probably do the same here. He seems to be absolutely good."—*New York Herald.*

